# Synthesis and Characterization of Cholesteryl Conjugated Lysozyme (CHLysozyme)

**DOI:** 10.3390/molecules25163704

**Published:** 2020-08-14

**Authors:** Shinji Katsura, Takayuki Furuishi, Haruhisa Ueda, Etsuo Yonemochi

**Affiliations:** 1 Department of Physical Chemistry, School of Pharmacy and Pharmaceutical Sciences, Hoshi University, 2-4-41 Ebara, Shinagawa-ku, Tokyo 142-8501, Japan; ds1855@hoshi.ac.jp (S.K.); t-furuishi@hoshi.ac.jp (T.F.); h.uedas23cd@jcom.zaq.ne.jp (H.U.); 2 Formulation research Lab., Taiho Pharmaceutical Co., Ltd., 224-2, Ebisuno, Hiraishi, Kawauchi-cho, Tokushima 771-0194, Japan

**Keywords:** lysozyme, cholesterol, conjugation, protein folding, hydrophobic interaction, enzyme activity, solution stability, resistance to organic solvents

## Abstract

Hydrophobic interaction is important for protein conformation. Conjugation of a hydrophobic group can introduce intermolecular hydrophobic contacts that can be contained within the molecule. It is possible that a strongly folded state can be formed in solution compared with the native state. In this study, we synthesized cholesteryl conjugated lysozyme (CHLysozyme) using lysozyme and cholesterol as the model protein and hydrophobic group, respectively. Cholesteryl conjugation to lysozyme was confirmed by nuclear-magnetic resonance. Differential-scanning calorimetry suggested that CHLysozyme was folded in solution. CHLysozyme secondary structure was similar to lysozyme, although circular dichroism spectra indicated differences to the tertiary structure. Fluorescence measurements revealed a significant increase in the hydrophobic surface of CHLysozyme compared with that of lysozyme; CHLysozyme self-associated by hydrophobic interaction of the conjugated cholesterol but the hydrophobic surface of CHLysozyme decreased with time. The results suggested that hydrophobic interaction changed from intramolecular interaction to an intermolecular interaction. Furthermore, the relative activity of CHLysozyme to lysozyme increased with time. Therefore, CHLysozyme likely forms a folded state with an extended durability of activity. Moreover, lysozyme was denatured in 100% DMSO but the local environment of tryptophan in CHLysozyme was similar to that of a native lysozyme. Thus, this study suggests that protein solution stability and resistance to organic solvents may be improved by conjugation of a hydrophobic group.

## 1. Introduction

Recently, the chemical modification of proteins for improvement of functionality has become widespread with advances in organic synthetic chemistry and protein engineering [1,2]. There are several ways to chemically modify proteins, for instance conjugating polyethylene glycol to protein (PEGylated protein) is of value as this enhances the chemical stability, solubility, and blood retention of the protein during clinical use [3,4,5,6,7,8]. The chemical stability (e.g., against thermal stress) of proteins has also been improved by modification with polysaccharides, which are hydrophilic groups [9,10,11]. The chemical modification of proteins has mostly been via conjugation with hydrophilic groups, but there are a few reports on chemical modification using hydrophobic groups [12,13]. Chemical modification of hydrophobic groups to water soluble polymers has been reported. For example, Akiyoshi et al. used hydrogel biomaterials such as cholesterol-bearing pullulan (CHP) [14], cholesterol-bearing poly-L-lysine (CHPLL) [15], cholesterol-bearing hydroxypropyl cellulose [16], and cholesterol-bearing xyloglucan [17].

These cholesterol-bearing materials self-assemble to form nanogels through hydrophobic interactions of the cholesteryl groups which were conjugated in the molecule in water. Moreover, CHP forms a complex with denatured protein by hydrophobic interaction with the cholesterol; the denatured protein is subsequently refolded in CHP, which has a function similar to a natural molecular chaperone [18,19]. Additionally, CHP has potential applications as a drug delivery system due to the ability to trap hydrophobic drugs [20]. In CHPLL and maltopentase-substituted CHPLL [21], hydrophobic interaction by cholesterol induces the formation of α-helical structures. Therefore, it is expected that chemical modification of hydrophobic groups to water soluble polymers can improve functionality and add novel functions by inducing inter/intramolecular hydrophobic interaction.

Chemical modification of hydrophobic groups to proteins have not been reported to the same degree. In native proteins, the hydrophobic residues are internally situated [22,23], although these residues can be exposed to the surface if the protein is denatured by chemical or physical stress [24,25]; moreover, protein aggregation has been assumed to be due to hydrophobic interaction between denatured proteins with surface-exposed hydrophobic residues [26,27]. Furthermore, aggregation affects biological activity and causes immunogenicity, and thus must be controlled during development of biopharmaceuticals [28,29]. Therefore, few reports have studied chemically modifying hydrophobic groups to proteins because it is assumed that conjugation with hydrophobic groups accelerates protein aggregation.

However, conjugation of a hydrophobic group into a protein can introduce intermolecular hydrophobic contacts that can be contained within the molecule. Therefore, it is possible that a strongly folded state can be formed in solution compared with the native state. Consequently, protein stability in solution, in both aqueous and organic solvents, can be improved; such improvements are expected to have pharmaceutical applications, for instance in protein formulation (using a liquid formulation as dosage form). Furthermore, improved resistance to organic solvents would enable further chemical modification of the hydrophobic conjugated protein.

Lysozyme (hen egg white lysozyme) is a single-chain protein consisting of 129 amino acids with a molecular weight of 14.3 kDa [30], whose structure, physicochemical properties, and folding pathway are well understood. Lysozyme has been widely used in pharmaceutical manufacturing and food preparation because of the lytic activity against cell walls of various Gram-positive bacteria [30,31]. Moreover, lysozyme is a small protein, but has lytic activity to large substrates, such as peptidoglycan in bacterial cell walls, and represents a model for other small proteins [32,33]. Therefore, we used lysozyme as a model protein. Cholesterol has been extensively used to enhance the hydrophobicity, biocompatibility, and biodegradability of drug delivery systems to avoid using slowly degrading high molecular weight polymers [34]. Cholesterol is an indispensable structural building block for cell membranes, and is responsible for membrane fluidity and permeability, intracellular transport, signal transduction, and cell trafficking [35,36,37].

In this study, we synthesized cholesteryl conjugated lysozyme (CHLysozyme) to improve lysozyme functionality. We also investigated the structure of CHLysozyme using nuclear-magnetic resonance (NMR) spectroscopy, differential-scanning calorimetry (DSC), circular dichroism (CD), and fluorescence measurement. Surface hydrophobicity of CHLysozyme was also evaluated by fluorescence measurement using 8-anilinonaphthalene-1-sulfonic acid ammonium salt (ANS). Furthermore, novel functionality, such as improvement of stability in buffer solution was evaluated using the enzyme activity of *Micrococcus lysodeikticus* (*M.l.*), and we investigated CHLysozyme stability against dimethyl sulfoxide (DMSO) using fluorescence measurement. The enhanced stability of CHLysozyme compared with that of lysozyme suggests that the addition of hydrophobic moieties, such as cholesterol, to proteins may have general implications for improvement of protein stability, particularly when further modifications or techniques involving organic solvents are required.

## 2. Results and Discussion

### 2.1. Confirmation of Conjugating the Cholesterol to Lysozyme by NMR Spectroscopy

**Figure 1:**
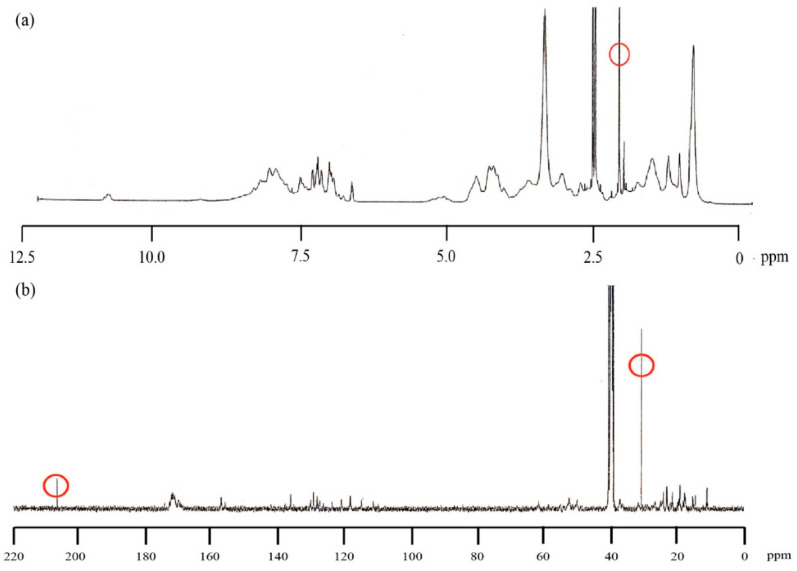
^1^H Nuclear-magnetic resonance (NMR) and ^13^C NMR spectra of CHLysozyme in DMSO-d_6_ at 25 °C. (**a**) ^1^H NMR spectrum, and (**b**) ^13^C NMR spectrum, CHLysozyme concentration: 21.4 mg/mL.

We used NMR to confirm the conjugation of cholesterol to lysozyme. Figure 1 shows the ^1^H NMR and ^13^C NMR spectra of cholesteryl conjugated lysozyme (CHLysozyme). We also measured the ^1^H NMR of lysozyme alone and of a physical mixture of lysozyme and cholesterol (lysozyme [mol]/cholesterol [mol] = 1/12) (Figure S1). From this data, we confirmed that the ^1^H NMR spectrum of CHLysozyme differed from the physical mixture. Akiyoshi et al. reported that ^1^H signal of the cholesteryl group of CHP (0.6–2.4 ppm) was clearly apparent in dimethyl sulfoxide (DMSO)-d_6_ [14]. Thus, we assumed that ^1^H signal at approximately 2 ppm in CHLysozyme was derived from cholesterol as this signal was not observed in lysozyme or the physical mixture. The ^13^C NMR spectrum suggested that the signals at approximately 30 ppm and 200 ppm in CHLysozyme are derived from carbon in the cholesteryl group and the carbonyl carbon (C=O) that connects lysozyme to cholesterol as those signals were not observed in lysozyme (Figure S2). Furthermore, ^1^H-^13^C Heteronuclear Multiple Quantum Correlation (HMQC) NMR measurement demonstrated a correlation between the ^1^H signal at ~2.0 ppm and the ^13^C signal at ~30 ppm (Figure S3). Therefore, we successfully synthesized lysozyme conjugated with cholesterol.

### 2.2. Evaluation of Protein State and Hydrophobic Residues in CHLysozyme by DSC

**Figure 2:**
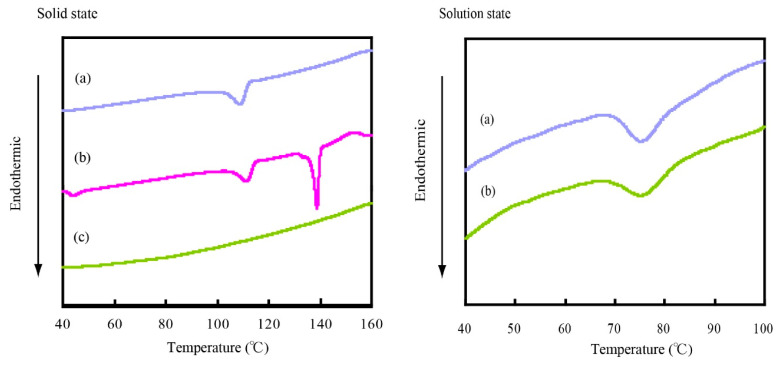
Differential-scanning calorimetry (DSC) thermograms of each lysozyme in solid state (left) and solution state (right). Solid state: (**a**) lysozyme, (**b**) physical mixture of lysozyme and cholesterol (lysozyme [mol]/cholesterol [mol] = 1/12) and (**c**) CHLysozyme. Weight of each sample: 5 mg. Solution state: (**a**) lysozyme, and (**b**) CHLysozyme. Lysozyme and CHLysozyme concentration: 30 mg/mL.

Protein is denatured under thermal stress and undergoes conformational collapse. DSC is used to study the thermal stability of proteins based on the detection of conformational changes in their structure, provided by measurement of the endothermic peak. The endothermic peak indicates a cooperative structural transition, which is in the case of the proteins is typically a native-denatured transition, both in solid [38,39,40] and solution [41,42,43,44]. Moreover, the change from native (folded) to the denatured (unfolded) structure causes the increase in hydrophobic hydration. To confirm the state of hydrophobic residues in CHLysozyme, we carried out the DSC measurement in solid state and in 0.05 mol/L phosphate buffer (pH 6.5). Figure 2 shows the DSC thermograms of lysozyme and CHLysozyme in solid (left) and in buffer solution (i.e., solution state) (right).

In solid state (Figure 2, left plot), DSC thermograms for lysozyme have a maximum peak of the endothermic point (*Tm*) at 109 °C, whereas the physical mixture between lysozyme and cholesterol had a *Tm* at 111 °C and 139 °C, respectively. In contrast, an endothermic peak for CHLysozyme was not observed in the measurement range. DMSO and acetone were used in the synthesis process of CHLysozyme. Therefore, we believe that CHLysozyme was denatured by these solvents and the endothermic peak was not observed.

In solution state (Figure 2, right plot), the *Tm* of both lysozyme and CHLysozyme were observed at 75 °C. The results suggested that CHLysozyme is folded in solution state. Akiyoshi et al. reported that CHPLL [15] formed hydrogel nanoparticles by self-association derived from the hydrophobic interaction of cholesteryl group. In CHPLL and maltopentase-substituted CHPLL [21], hydrophobic interaction by cholesteryl group induced the formation of an α-helical structure. Therefore, it is possible α-helical structure is induced in CHLysozyme by conjugated cholesterol.

### 2.3. Evaluation of the Secondary and Tertiary Structure of CHLysozyme by CD Measurement

According to DSC measurements, CHLysozyme was denatured in solid state but remained folded in solution. Thus, we considered that CHLysozyme has a secondary structure. CD measurement is an effective tool for determination of the secondary structure of a protein [45,46].

Lysozyme (α+β type) protein is composed of both α-helix and β-sheet secondary structures with a larger content of α-helix than that of β-sheet [47]. CD measurement in the far UV region (200–250 nm) provides information on protein secondary structures. The ellipticity at 222 nm in the CD spectrum also reflects the α-helical content of a protein and its absolute value increases with increasing α-helical content [45,46,48].

**Figure 3:**
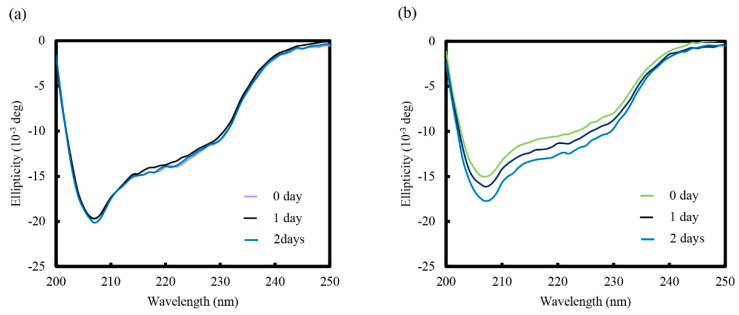
Change of circular dichroism (CD) spectra of lysozyme (**a**) and CHLysozyme (**b**) with time in the far UV (200 nm to 250 nm) region at 25 °C. Lysozyme and CHLysozyme concentration: 0.2 mg/mL.

CD measurement in the far UV region (200–250 nm) suggested that the secondary structure of CHLysozyme was composed of α-helices and β-sheets. The CD spectrum was very similar to that of lysozyme (Figure 3a,b), but the absolute value of CHLysozyme at 222 nm was lower than that of lysozyme, indicating that the α-helical content of CHLysozyme was also lower than that of lysozyme. Previous reports indicated that physical energy such as agitation and heating over five hours was needed to form the hydrogel nanoparticles in CHP and CHPLL [14,15]. We avoided this operation, because there was a possibility that the protein would denature due to thermal stress and physical energy. CHPLL forms an α-helical structure by hydrophobic interaction with cholesterol [15]. Therefore, it is possible that CHLysozyme also forms an α-helical structure selectively by hydrophobic interaction with cholesterol. Consequently, we assessed the change of CD spectra in the far UV region of CHLysozyme over time and confirmed the changes in the secondary structure of CHLysozyme. Figure 3 demonstrates the shifts within the far UV region of the CD spectra for lysozyme and CHLysozyme with increasing time. The absolute value of CHLysozyme at 222 nm (α-helical content of CHLysozyme) increased with time (Figure 3b); in contrast, the lysozyme CD spectrum did not change (Figure 3a). An α-helical structure is formed by hydrogen bonding between amino groups and carboxyl groups in protein, and we therefore considered that the cholesteryl group of CHLysozyme could interact with hydrophobic residues, such as aromatic amino acids, in the CHLysozyme molecule. Formation of a folded state of CHLysozyme, via interacting cholesteryl groups and hydrophobic residues of CHLysozyme within the molecule, could reduce the distance between these amino acids and the carboxyl group and accelerate formation of α-helical structure. The CD spectra suggested that α-helical content (ellipticity at 222 nm) of CHLysozyme increased over time. Therefore, we considered that CHLysozyme formed a folded state by hydrophobic interaction of the cholesteryl group and hydrophobic residues of CHLysozyme within the molecule.

**Figure 4:**
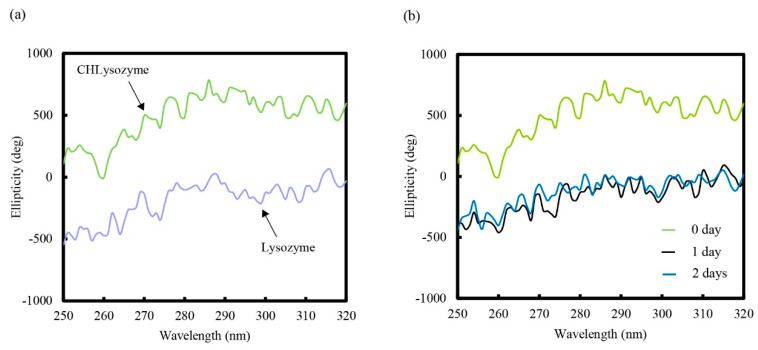
CD spectra of each lysozyme (**a**) and change of CD spectra of CHLysozyme with time (**b**) in the near UV (250 nm to 320 nm) region at 25 °C. Lysozyme and CHLysozyme concentration: 0.2 mg/mL.

CD spectra in the near UV region (250 nm to 320 nm) revealed the environment of aromatic amino acid residues and the tertiary structure of a protein [45,46]. Figure 4a shows that the CD spectra of lysozyme differed from that of CHLysozyme in the near UV region. This result indicated that the conformational change in lysozyme had occurred due to cholesterol conjugation. In addition, we evaluated the changing CD spectra of CHLysozyme with increasing time (Figure 4b). CD spectra in the near UV region changed after one day, but remained similar after a further day. This indicated that the secondary structure of CHLysozyme altered with increasing time, but the tertiary structure was not changed. Although hydrogen bonding is vital for secondary structure formation, hydrophobic interaction and disulfide bonds are important to form tertiary structure. From those results, we assumed that hydrophobic interaction of CHLysozyme changed from an intermolecular interaction, such as CHLysozyme-CHLysozyme interaction, to an intramolecular interaction, namely the hydrophobic interaction between hydrophobic amino acid residues and the cholesteryl group in CHLysozyme molecule after one day.

### 2.4. Evaluation of Particle Size for CHLysozyme

We measured the particle size of lysozyme and CHLysozyme to confirm whether intermolecular interaction was present. Table 1 shows the particle size of lysozyme and CHLysozyme with their increasing concentration. CHLysozyme had a larger particle size compared with that of lysozyme at each concentration indicating that CHLysozyme was self-associating in solution. Additionally, the increase in the particle size of CHLysozyme was concentration-dependent.

β-cyclodextrin (β-CyD) has a high binding constant to cholesterol and traps cholesterol in a hydrophobic cavity [49,50]. Moreover, a complex of horseradish peroxidase (HRP) was released from the CHP nanogel by the addition of β-CyD because of the interaction with the cholesteryl group of CHP [51]. Therefore, we hypothesized that if the driving force of association was intermolecular hydrophobic interaction concerning the CHLysozyme cholesteryl group, the particle size of CHLysozyme would decrease with β-CyD addition. We measured a decrease in the particle size of CHLysozyme (1.0 mg/mL) following the addition of β-CyD (Figure S4b). Thus, we concluded that the driving force to form association with CHLysozyme was mainly intermolecular hydrophobic interaction derived from the CHLysozyme cholesteryl group.

### 2.5. Investigation of the Environment Around Aromatic Amino Acid Residues of CHLysozyme by Fluorescence Measurement

The absorption and fluorescence emission at 280 nm indicated the environment around the aromatic amino acid residues, mainly tryptophan and tyrosine due to their high quantum yield and extinction coefficient [52,53], and thus fluorescence measurement could also be used to study protein structure [48,54].

**Figure 5:**
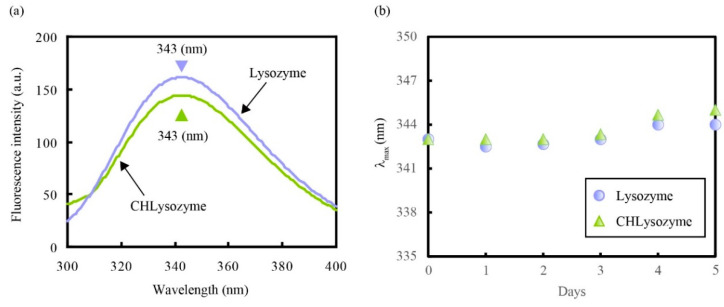
Fluorescence emission spectra (**a**) and λ_max_ change against time (**b**) of lysozyme and CHLysozyme at 25 °C. CHLysozyme concentration: 0.2 mg/mL. Excitation wavelength: 280 nm. Gain value of fluorescence spectrometer: medium.

Fluorescence emission spectrum is shifted to short wavelengths (blue shift) once the environment around aromatic acid residues of protein changes to a hydrophobic environment [53,54,55,56,57]. Conversely, there is a shift to longer wavelengths (red shift) when the environment around aromatic acid residues of protein changes to a hydrophilic environment [55]. Therefore, the native or denatured state of a protein can be evaluated based on those changes. Figure 5a shows the fluorescence spectra of lysozyme and CHLysozyme. Fluorescence emission spectra maximum (λ_max_) of lysozyme and CHLysozyme were observed at 343 nm. The λ_max_ of tryptophan is observed at around 340 nm, so the fluorescence emission spectra of both lysozyme and CHLysozyme were mainly attributed to tryptophan. This result indicated that the environment around the aromatic amino acid residues of CHLysozyme was highly similar to that of lysozyme. Furthermore, the λ_max_ of both lysozyme and CHLysozyme did not significantly change with increasing time (Figure 5b). Thus, we concluded that the aromatic amino acids (mainly tryptophan residues) present in the CHLysozyme molecule and their surrounding environment in CHLysozyme did not alter with increasing time.

### 2.6. Study of the Hydrophobic Region of CHLysozyme by Fluorescene Measurement with a Hydrophobic Probe

Particle size measurement suggested that CHLysozyme association occurred in solution. The driving force for this association was mainly intermolecular hydrophobic interaction between cholesteryl group-cholesteryl group and/or between cholesteryl group-hydrophobic residues in CHLysozyme. Conversely, if a hydrophobic interaction by cholesteryl group occurred within the CHLysozyme molecule then the CHLysozyme self-association would not occur. Therefore, we believed the cholesteryl group was conjugated at the surface of CHLysozyme. However, an increasing α-helical content with increasing time was observed by CD measurement. This phenomenon may be caused by a shift from hydrophobic interaction, such as intermolecular, to intramolecular after one day. Thus, surface hydrophobicity of CHLysozyme would be dependent on the location of the cholesteryl residue.

**Figure 6:**
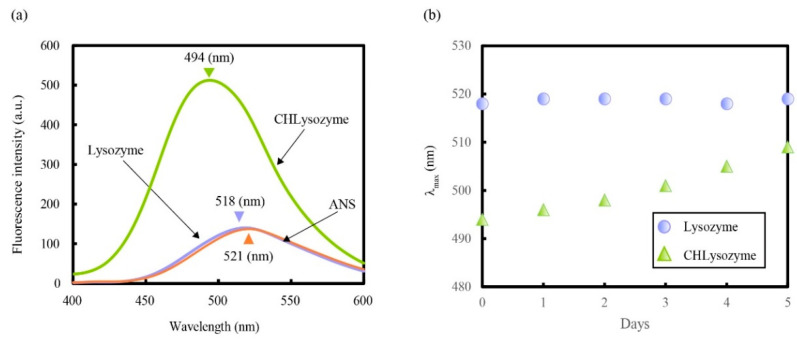
Fluorescence measurements of 8-anilinonaphthalene-1-sulfonic acid ammonium salt (ANS) alone and ANS with each lysozyme at 25 °C. (**a**) Fluorescence emission spectra of ANS alone and ANS with lysozyme and CHLysozyme at 25 °C. ANS concentration: 1.0 × 10^−4^ mol/L; lysozyme and CHLysozyme concentration: 0.2 mg/mL. Excitation wavelength: 365 nm. Gain value of fluorescence spectrometer: medium. (**b**) Change in λ_max_ of ANS with lysozyme and CHLysozyme against time at 25 °C. ANS concentration: 1.0 × 10^−4^ mol/L; lysozyme and CHLysozyme concentration: 0.2 mg/mL. Excitation wavelength: 365 nm. Gain value of fluorescence spectrometer: medium.

We measured fluorescence with excitation wavelength at 365 nm using 8-anilinonaphthalene-1-sulfonic acid ammonium salt (ANS) to study hydrophobicity on the surface of CHLysozyme. ANS has been widely used as a fluorescent probe for the characterization of protein binding sites and the study of folding pathways [58,59,60,61] as it binds to hydrophobic regions on protein surfaces with high affinity. The λ_max_ of ANS undergoes a blue shift to a shorter wavelength by transition from a polar to a nonpolar environment, and the fluorescence intensity is increased. The same phenomena is observed when ANS binds to a hydrophobic region on the surface of proteins. Figure 6a shows the fluorescence emission spectra of ANS alone and ANS with lysozyme and CHLysozyme. The λ_max_ exhibited a blue shift (521 nm to 494 nm) against the λ_max_ of ANS alone; the intensity of ANS was significantly increased in CHLysozyme, whereas λ_max_ of ANS was almost unchanged in lysozyme. This suggested that the hydrophobic region of CHLysozyme increased in comparison with that of lysozyme at a concentration of 0.2 mg/mL.

The increase in the hydrophobicity indicated that surface hydrophobicity of CHLysozyme was higher compared with that of lysozyme. Therefore, we assumed that this increase would be derived from the cholesteryl group on the surface of CHLysozyme. Once the cholesteryl group was exposed on the surface of CHLysozyme, hydrophobic interaction by these groups would easily occur between CHLysozyme molecules. The particle size of CHLysozyme at approximately 119 nm at 0.2 mg/mL suggested CHLysozyme self-association. Thus, we concluded that the cholesteryl group was present on the surface of CHLysozyme at 0.2 mg/mL.

Figure 6b shows the change in λ_max_ of ANS with lysozyme and CHLysozyme against time. The ANS λ_max_ with lysozyme did not change, but the λ_max_ of ANS with CHLysozyme gradually underwent a red shift to increasing wavelengths with time, indicating a decrease in hydrophobicity on the surface of CHLysozyme. Taking into consideration the CD measurements, we assumed that the hydrophobic interaction of CHLysozyme changed from intermolecular CHLysozyme-CHLysozyme interaction to intramolecular interactions, implying there were hydrophobic interactions between hydrophobic amino acid residues and the cholesteryl group in the CHLysozyme molecule. Thus, the lower shifting λ_max_ of ANS with CHLysozyme to longer wavelengths implied a decrease in hydrophobicity on the surface of CHLysozyme. Again, this suggested a change of hydrophobic interaction from intermolecular interaction to intramolecular interaction. Thus, we concluded that cholesteryl group of CHLysozyme was situated on the surface after dissolution in buffer solution, and that the hydrophobic interactions of the cholesteryl groups mainly occurred between CHLysozyme molecules. Moreover, we assumed that hydrophobic interaction by these groups dominantly changed to intramolecular with time based on the changes to λ_max_.

We also investigated the surface hydrophobicity of CHLysozyme at concentrations of 0.02 and 2.0 mg/mL. Figure S5a shows the fluorescence emission spectra of ANS alone and ANS with lysozyme and CHLysozyme at the concentration of 0.02 mg/mL. The λ_max_ of ANS with lysozyme or CHLysozyme was similar to that of ANS alone, suggesting that the cholesteryl group was folded inside CHLysozyme. Therefore, we assumed that the hydrophobic interaction by conjugated cholesterol was most significant within the molecule because the distance between molecules would be greater at lower concentrations compared to those at 0.2 mg/mL. Additionally, changes in λ_max_ of ANS with lysozyme and CHLysozyme against time were not observed (Figure S5b). Therefore, we concluded that the intramolecular interactions predominated with the cholesteryl group in CHLysozyme and hydrophobic residues in lysozyme, and that the cholesteryl group in CHLysozyme was not presented on the surface at lower concentrations. However, the concentration of CHLysozyme was low and it might be that the association constant between ANS and CHLysozyme was small.

Increasing the enzyme concentration to 2.0 mg/mL resulted in a blue shift of λ_max_ of ANS with lysozyme and CHLysozyme compared to that of ANS alone, and a large change in λ_max_ of ANS with CHLysozyme compared with that of lysozyme (Figure S6a). The change in λ_max_ of ANS with CHLysozyme was also larger compared with that of ANS at 0.2 mg/mL. At higher concentrations, the distance between CHLysozyme molecules is reduced, and hydrophobic interaction between these would easily occur via the cholesteryl group of the CHLysozyme surface. Thus, larger associations would be formed by intermolecular hydrophobic interaction between the cholesteryl group and/or hydrophobic residues of CHLysozyme. The λ_max_ of ANS with lysozyme and CHLysozyme also remained constant over time (Figure S6b). Thus, the hydrophobic interaction by conjugated cholesterol is intermolecular at higher concentration.

**Figure 7:**
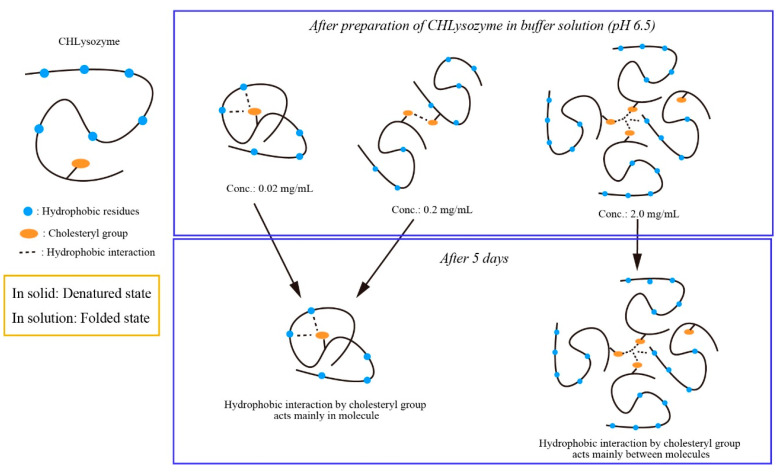
Possible scheme for hydrophobic interaction of CHLysozyme at concentrations tested in the study.

Figure 7 depicts a schematic of the possible hydrophobic interactions of CHLysozyme at each concentration. We concluded that hydrophobic interaction by the cholesteryl group mainly behaved as intramolecular at 0.02 and 0.2 mg/mL, whereas hydrophobic interaction by the cholesteryl group dominantly was mainly intermolecular at higher concentrations.

### 2.7. Enzyme Activity of CHLysozyme

#### 2.7.1. Relative Activity and Enzyme Kinetic Constants of CHLysozyme

CD measurements suggested that the secondary structure of CHLysozyme was similar to lysozyme (α+β type structure), but that the tertiary structure differed between the two enzymes. Therefore, the conformational change induced by the cholesteryl group may affect the enzyme activity of CHLysozyme compared with that of lysozyme. Hence, we evaluated the enzyme activity of lysozyme and CHLysozyme using *Micrococcus lysodeikticus* (*M.l.*) as substrate by monitoring the turbidity at 450 nm [62]. This demonstrated that the relative activity of CHLysozyme to lysozyme was approximately 95%, implying that CHLysozyme had maintained a similar enzyme activity as lysozyme.

We then measured the enzyme activity of lysozyme and CHLysozyme to *M.l.* (0.02–0.2 mg/mL) at 25 °C and calculated the Michaelis constant (K_m_) and maximum rate (V_max_) from Lineweaver-Burk plots (Figure S7 and Table 2). The K_m_ and V_max_ of CHLysozyme were lower than that of lysozyme. The K_m_ value indicates the affinity of an enzyme to the substrate; a smaller K_m_ suggests that the enzyme-substrate complex forms at lower concentrations. The V_max_ indicates the catalytic efficiency of the enzyme. Thus, we concluded that the affinity of CHLysozyme to the substrate was improved by conformational change induced by cholesteryl conjugation to lysozyme.

#### 2.7.2. Change of Relative and Residual Activity over Time

We demonstrated that hydrophobic interaction mediated by the cholesteryl group in CHLysozyme gradually changed from intermolecular to intramolecular. We therefore assumed that CHLysozyme formed a folded state by hydrophobic interactions of the cholesteryl group with the hydrophobic residues of CHLysozyme, and this was expected to improve the solution stability. Therefore, we evaluated the change of the relative and residual activity of lysozyme and CHLysozyme with increasing time.

**Figure 8:**
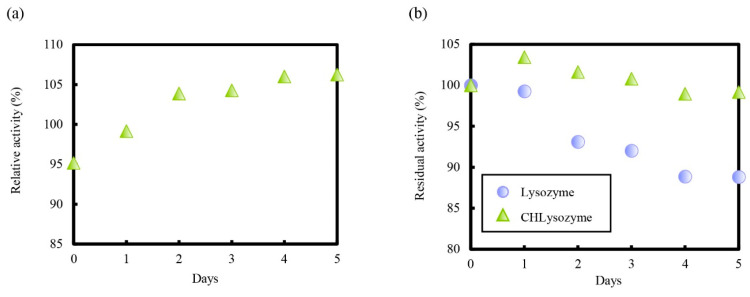
Enzyme activity of CHLysozyme in 0.05 mol/L phosphate buffer (pH 6.5) at 25 °C. (**a**) Relative activity of CHLysozyme to lysozyme, (**b**) residual activity of lysozyme and CHLysozyme. Lysozyme and CHLysozyme concentration: 0.2 mg/mL; *Micrococcus lysodeikticus* concentration: 0.16 mg/mL.

Figure 8a shows the relative activity of CHLysozyme to lysozyme at 0.05 mol/L in a phosphate buffer (pH 6.5) at 25 °C over time. The relative activity of CHLysozyme increased over time and reached over 100% after two days. We checked the residual activity of each lysozyme plotted against time (Figure 8b). The residual activity of lysozyme and CHLysozyme decreased with increasing days, although the residual activity of CHLysozyme remained superior to that of lysozyme throughout this time. This suggested that CHLysozyme formed a folded state by hydrophobic interaction of the cholesteryl group as an intramolecular interaction, and we concluded that residual activity of CHLysozyme in solution was improved compared with that of lysozyme.

K_m_ of CHLysozyme was smaller than that of lysozyme (Table 2), and decreasing K_m_ would be induced by conformational change of cholesteryl conjugation to lysozyme. CD spectra of CHLysozyme in the near UV region (250–320 nm) changed after one day, but remained similar after a further day (Figure 4b). It is possible that the tertiary structure of CHLysozyme was different to lysozyme after one day and we investigated the CD spectra of lysozyme and CHLysozyme in the near UV region (250–320 nm) after two days (Figure S8). The results suggested that the tertiary structure of CHLysozyme was different to lysozyme. Therefore, we assumed that improvement of residual activity may be caused by conformational change in addition to formation of folded state by hydrophobic interaction of the cholesteryl group.

### 2.8. Resistance to DMSO of CHLysozyme

Hattacharjya and Balaram reported that lysozyme formed a partially folded state with increasing DMSO concentration (>10%), and this transition was essentially completed by ~50% concentration [63,64]. Furthermore, a highly unfolded state of lysozyme was reported with 100% DMSO [65]. In solid state, the endothermic peak of CHLysozyme by DSC was not observed, and we considered that CHLysozyme was unfolded in solid state as 100% DMSO had been used in the synthesis of CHLysozyme. However, in buffer solution, CHLysozyme was folded and not only had similar activity to lysozyme but also had superior residual activity. Therefore, we assumed that CHLysozyme formed a folded state in solution. Therefore, we assumed that CHLysozyme may form a folded state in organic solvents. Local environment around tryptophan moieties of CHLysozyme in DMSO can be evaluated by fluorescence measurement and this result might be as an indication of folding state. Thus, we carried out the fluorescence measurement at 25 °C to evaluate the local environment around tryptophan moieties of CHLysozyme in DMSO.

**Figure 9:**
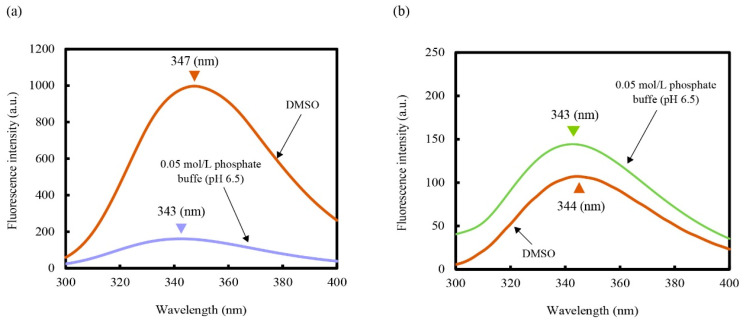
Fluorescence emission spectra of lysozyme (**a**) and CHLysozyme (**b**) in 0.05 mol/L phosphate buffer (pH 6.5) and DMSO at 25 °C. Lysozyme and CHLysozyme concentration: 0.2 mg/mL. Excitation wavelength: 280 nm. Gain value of fluorescence spectrometer: medium.

We measured the fluorescence of lysozyme and CHLysozyme at the excitation wavelength of 280 nm in 0.05 mol/L phosphate buffer (pH 6.5) and 100% DMSO at 25 °C (Figure 9). The λ_max_ of lysozyme in DMSO (347 nm) exhibited a red shift against that of lysozyme in 0.05 mol/L phosphate buffer (pH 6.5, 343 nm) with a substantial increase in fluorescence. Conversely, the λ_max_ of CHLysozyme in DMSO (344 nm) was highly similar to that in 0.05 mol/L phosphate buffer (pH 6.5); fluorescence intensity of CHLysozyme in DMSO was slightly decreased compared with that in 0.05 mol/L phosphate buffer (pH 6.5). The red shift of λ_max_ implied the environment around the aromatic acid residues had changed to a hydrophilic environment that would expose aromatic amino acid residues at the protein surface [53,54,55,56,57]. Fluorescence intensity was also increased by exposure of aromatic amino acids at the protein surface. These results suggested that lysozyme was denatured in 100% DMSO but that the local environment of tryptophan moieties in CHLysozyme was similar to that of a native lysozyme. Thus, we assumed that CHLysozyme may have resistance to DMSO.

## 3. Materials and Methods

### 3.1. Materials

Hen egg white lysozyme (lysozyme), *Micrococcus lysodeikticus* (*M.l.*), and 8-anilinonaphthalene-1-sulfonic acid ammonium salt (ANS) were purchased from SIGMA Aldrich (St. LA, USA). Cholesterol, *N,N*′-carbonyldiimidazole (CDI), dimethyl sulfoxide (DMSO), dehydrated DMSO, DMSO-d_6_, and acetone were purchased from FUJIFILM Wako Pure Chemical Industries, Ltd. (Osaka, Japan); β-cyclodextrin (β-CyD) was purchased from Nihon Shokuhin Kako Co., Ltd. (Tokyo, Japan). All other reagents were commercial products of analytical grade.

### 3.2. Synthesis of Cholesteryl Conjugated Lysozyme (CHLysozyme)

CDI is used as an amino acid coupling agent and introduces a chelated residue to a water-soluble polymer [66,67,68,69]. Imidazole in CDI has high reactivity and it can be converted to an ester via a reaction with alcohol and amine.

For conjugation to lysozyme, cholesterol was agitated with CDI in dehydrated DMSO at 50 °C for 3 h and cooled to room temperature; lysozyme was then added to the following ratio: CDI [mol]/lysozyme [mol] = 10 and cholesterol [mol]/lysozyme [mol] = 12. The solution was agitated at room temperature for 20 h to conjugate the cholesteryl group to the hydroxyl and amino groups of lysozyme. CHLysozyme powder was obtained by adding acetone, washing with acetone several times and drying under reduced pressure at room temperature, and then storing under-20 °C (Figure S9).

### 3.3. Confirmation of Conjugating the Cholesterol to Lysozyme by NMR Spectroscopy

Lysozyme (15 mg) and CHLysozyme (15 mg) were separately dissolved in DMSO-d_6_. The solutions were filtered through a membrane filter (0.45 μm) and were analyzed by NMR spectrometer (JNM-LA500, JEOL, Tokyo, Japan). NMR (500 MHz ^1^H-, 125 MHz ^13^C-, ^1^H-^13^C HMQC) spectra were recorded at 25 °C. DMSO (2.49 ppm) was used as an internal standard in DMSO-d_6_.

### 3.4. Evaluation of Protein State and Hydrophobic Residues in CHLysozyme by DSC

In solid state, lysozyme (5 mg), a physical mixture of lysozyme and cholesterol (lysozyme [mol]/cholesterol [mol] = 1/12) (5 mg), and CHLysozyme (5 mg) were analyzed by DSC (DSC8240D, Rigaku, Tokyo, Japan). The scan rate and scan range were set at 2 °C/min and 40–160 °C, respectively; Alumina (Al_2_O_3_) was used as a reference material.

In solution state, lysozyme (30 mg/mL) and CHLysozyme solution (30 mg/mL) were separately prepared in 0.05 mol/L phosphate buffer (pH 6.5). The solutions were analyzed by DSC (DSC8240D). The scan rate and scan range were set at 2 °C/min and 40–100 °C, respectively; 0.05 mol/L phosphate buffer was used as a reference material.

### 3.5. Evaluation of the Secondary and Tertiary Structures of CHLysozyme by CD Measurement

Lysozyme and CHLysozyme were separately dissolved in 0.05 mol/L phosphate buffer (pH 6.5) to 0.2 mg/mL. The solutions were analyzed by CD spectrometer (J-820, JASCO, Tokyo, Japan). CD spectra were recorded from 200 to 320 nm at 25 °C.

### 3.6. Evaluation of Particle Size for CHLysozyme

Lysozyme and CHLysozyme were separately dissolved in 0.05 mol/L phosphate buffer (pH 6.5) to 0.2, 1.0, and 2.0 mg/mL. The solutions were analyzed by particle size analyzer (N5, BECKMAN COULTER, Tokyo, Japan). Particle size distributions were recorded at 25 °C.

### 3.7. Investigation of the Environment Around Aromatic Amino Acid Residues of CHLysozyme by Fluorescence Measurement

Lysozyme and CHLysozyme were separately dissolved in 0.05 mol/L phosphate buffer (pH 6.5) to 0.2 mg/mL. The solutions were analyzed by fluorescence spectrometer (FP-750, JASCO, Tokyo, Japan). The excitation wavelength was fixed at 280 nm with a band pass of 10 nm and emission band pass was set as 10 nm. Fluorescence emission spectra were recorded from 300 to 400 nm at 25 °C.

### 3.8. Study of the Hydrophobic Region of CHLysozyme by Fluorescence Measurement with a Hydrophobic Probe

Lysozyme, CHLysozyme, and ANS were separately dissolved in 0.05 mol/L phosphate buffer (pH 6.5). Each lysozyme and ANS solution were mixed to 0.02, 0.2, and 2.0 mg/mL and 1.0 × 10^−4^ mol/L, respectively. The solutions were analyzed by fluorescence spectrometer. The excitation wavelength was fixed at 365 nm with a band pass of 10 nm and emission band pass was set as 10 nm. Fluorescence emission spectra were recorded from 400 to 600 nm at 25 °C.

### 3.9. Enzyme Activity of CHLysozyme

#### 3.9.1. Relative Activity and Enzyme Kinetic Constants of CHLysozyme

##### Relative Activity of CHLysozyme to Lysozyme

Lysozyme and CHLysozyme were separately dissolved in 0.05 mol/L phosphate buffer (pH 6.5) to 0.2 mg/mL (enzyme solution). *M.l*. was dissolved in 0.05 mol/L phosphate buffer (pH 6.5) to 0.16 mg/mL (substrate solution). A measure of 0.3 mL of enzyme solution (3 h post-preparation) was added to 2.7 mL of substrate solution and the change of absorbance at 450 nm at 25 °C was analyzed by ultraviolet (UV) spectrometer (V-560, JASCO, Tokyo, Japan) for 10 sec. The relative activity of CHLysozyme was calculated by the formula:

Relative activity of CHLysozyme = (Initial reaction rate of CHLysozyme)/(Initial reaction rate of lysozyme) × 100

##### Enzyme Kinetic Constants of CHLysozyme

Lysozyme and CHLysozyme prepared as described for relative activity, except *M.l.* substrate solution was prepared from 0.04 to 0.20 mg/mL. The enzyme solution and substrate solution were mixed and the change of absorbance at 450 nm recorded as for relative activity. Initial reaction rate of each enzyme was calculated using initial slope. Michaelis constant (K_m_) and maximum rate (V_max_) of each enzyme were calculated using the Lineweaver-Burk plot.

#### 3.9.2. Change of Relative and Residual Activity over Time

Lysozyme and CHLysozyme were prepared as described for relative activity. Measurements were performed over five days using the same enzyme solutions, and each enzyme solution was stored at 25 °C. The relative activity for CHLysozyme was calculated as described above. The residual activity for both enzymes was calculated by the following formula:

Residual activity of each lysozyme = (Initial reaction rate of each lysozyme on measuring day)/(Initial reaction rate of each lysozyme on initial day) × 100

### 3.10. Resistance to DMSO of CHLysozyme

Lysozyme and CHLysozyme were separately dissolved in DMSO to 0.2 mg/mL. The solutions were analyzed by fluorescence spectrometer. The excitation wavelength was fixed at 280 nm with a band pass of 10 nm and emission band pass was set as 10 nm. Fluorescence emission spectra were recorded from 300 to 400 nm at 25 °C.

## 4. Conclusions

We succeeded in synthesizing cholesteryl conjugated lysozyme (CHLysozyme) using *N,N*′-carbonyldiimidazole (CDI) in dehydrated dimethyl sulfoxide (DMSO) and confirmed the conjugation of cholesterol to lysozyme through the carbonyl carbon (C=O) of CDI via NMR. We then characterized the structure of CHLysozyme and evaluated the effects of conjugation on enzyme activity.

CHLysozyme formed a folded state in buffer solution in comparison to solid unfolded state in solid based on DSC measurement. CD measurements suggested that the secondary structure of CHLysozyme was similar to lysozyme, although the tertiary structure differed between the two enzymes. Furthermore, fluorescence measurements using 8-anilinonaphthalene-1-sulfonic acid ammonium salt (ANS) indicated that the hydrophobic region on the surface of CHLysozyme was significantly increased compared with that of lysozyme. We considered that the driving force to form association with CHLysozyme was mainly intermolecular hydrophobic interaction derived from the cholesteryl group of CHLysozyme based on particle size measurements before and after addition of β-cyclodextrin (β-CyD). However, the λ_max_ of ANS obtained by fluorescence measurements gradually shifted to longer wavelengths with increasing time, suggesting that the hydrophobic region on the surface of CHLysozyme decreased with increasing time. We believe that hydrophobic interaction by the cholesteryl group changed from intermolecular interaction to intramolecular interaction. Furthermore, the relative activity of CHLysozyme to lysozyme increased over time, reaching over 100% after two days. Therefore, we concluded that CHLysozyme forms a folded state by the hydrophobic interaction of the cholesteryl group in the molecule with improvement to the residual activity of the enzyme. Additionally, lysozyme was denatured in 100% DMSO but the local environment of tryptophan in CHLysozyme was similar to that of a native lysozyme.

This study suggests that solution stability and resistance to organic solvents can be improved by addition of a hydrophobic group to the interior of a protein and by the subsequent hydrophobic interactions within the molecule. Conjugating proteins with cholesterol would increase protein stability not only in aqueous solution but also in organic solvents. Therefore, this technique has potential to modernize pharmaceutical techniques such as immobilized enzyme and protein formulation.

## Figures and Tables

**Table 1:** Particle size of lysozyme and CHLysozyme with their increasing concentration.

Sample	Conc. (mg/mL)	Particle Size (nm)
Lysozyme	0.2	N. D. ^1^
	1.0	24
	2.0	75
CHLysozyme	0.2	119
	1.0	238
	2.0	1194

^1^ N. D.: not detected.

**Table 2:** K_m_ and V_max_ of lysozyme and CHLysozyme calculated from Lineweaver-Burk plot.

Sample ^1^	K_m_ (mg/mL)	V_max_ (sec^−1^)
Lysozyme	1.20	0.07
CHLysozyme	0.25	0.02

^1^ Lysozyme and CHLysozyme concentration: 0.2 mg/mL; *Micrococcus lysodeikticus* concentration: 0.04–0.2 mg/mL.

## Supplementary Materials

The following are available online. Figure S1: ^1^H NMR spectra of (a) lysozyme, (b) CHLysozyme, and (c) physical mixture of lysozyme and cholesterol. Lysozyme and CHLysozyme concentration: 21.4 mg/mL; physical mixture of lysozyme and cholesterol (lysozyme [mol]/cholesterol [mol] = 1/12) concentration: 21.4 mg/m, Figure S2: ^13^C NMR spectra of lysozyme and CHLysozyme in DMSO-d_6_ at 25 °C. (a) Lysozyme, and (b) CHLysozyme. Lysozyme and CHLysozyme concentration: 21.4 mg/mL, Figure S3: ^1^H-^13^C HMQC NMR spectrum of CHLysozyme in DMSO-d_6_ at 25 °C. CHLysozyme concentration: 21.4 mg/mL, Figure S4: Particle size distribution of each lysozyme (a) and that of CHLysozyme before and after addition of β-cyclodextrin (β-CyD) (b). Lysozyme and CHLysozyme concentration: 1.0 mg/mL; β-CyD concentration: 0.2 × 10^−3^ mol/L. Preparation of the solution spiked with β-CyD: 0.1 mL of 0.2 × 10^−3^ mol/L β-CyD was added to 3.0 mL of CHLysozyme solution and the solution was analyzed 3 h post-preparation, Figure S5: Fluorescence measurements of ANS alone and ANS with each lysozyme at 25 °C. (a) Fluorescence emission spectra of ANS alone and ANS with lysozyme and CHLysozyme at 25 °C. ANS concentration: 1.0 × 10^−4^ mol/L; lysozyme and CHLysozyme concentration: 0.02 mg/mL. Excitation wavelength: 365 nm. Gain value of fluorescence spectrometer: medium, (b) Change in λ_max_ of ANS with lysozyme and CHLysozyme against time at 25 °C. ANS concentration: 1.0 × 10^−4^ mol/L; lysozyme and CHLysozyme concentration: 0.02 mg/mL. Excitation wavelength: 365 nm. Gain value of fluorescence spectrometer: medium, Figure S6: Fluorescence measurements of ANS alone and ANS with each lysozyme at 25 °C. (a) Fluorescence emission spectra of ANS alone and ANS with lysozyme and CHLysozyme at 25 °C. ANS concentration: 1.0 × 10^−4^ mol/L; lysozyme and CHLysozyme concentration: 2.0 mg/mL. Excitation wavelength: 365 nm. Gain value of fluorescence spectrometer: low, (b) Change in λ_max_ of ANS with lysozyme and CHLysozyme against time at 25 °C. ANS concentration: 1.0 × 10^−4^ mol/L; lysozyme and CHLysozyme concentration: 2.0 mg/mL. Excitation wavelength: 365 nm, Gain value of fluorescence spectrometer: low, Figure S7: Lineweaver−Burk plots of lysozyme and CHLysozyme at 25 °C. Lysozyme and CHLysozyme concentration: 0.2 mg/mL; *Micrococcus lysodeikticus* concentration: 0.04–0.2 mg/mL, Figure S8: CD spectra of each lysozyme in near UV (250 to 320 nm) region at 25 °C after two days. Lysozyme and CHLysozyme concentration: 0.2 mg/mL, Figure S9: Synthetic procedure for CHLysozyme.
