# Supplementary material for: Synthesis and Characterization of Cholesteryl Conjugated Lysozyme (CHLysozyme)
Source: Molecules. 2020 Aug 14;25(16):3704. doi: 10.3390/molecules25163704 (PMC7465789; doi:10.3390/molecules25163704)
Supplement: Supplementary file 1 [file molecules-25-03704-s001.pdf]

molecules
Supporting Materials

Synthesis and Characterization of Cholesteryl Conjugated Lysozyme (CHLysozyme)

Shinji Katsura [1,2], Takayuki Furuishi [1], Haruhisa Ueda [1] and Etuso Yonemochi [1,*]

[1] Department of Physical Chemistry, School of Pharmacy and Pharmaceutical Sciences, Hoshi University, 2-4-41 Ebara, Shinagawa-ku, Tokyo, 142-8501, Japan
[2] Formulation research Lab., Taiho Pharmaceutical Co., Ltd., 224-2, Ebisuno, Hiraishi, Kawauchi-cho, Tokushima, 771-0194, Japan
*Correspondence: e-yonemochi@hoshi.ac.jp; Tel.: +81-3-5498-5048 (Y.E.)

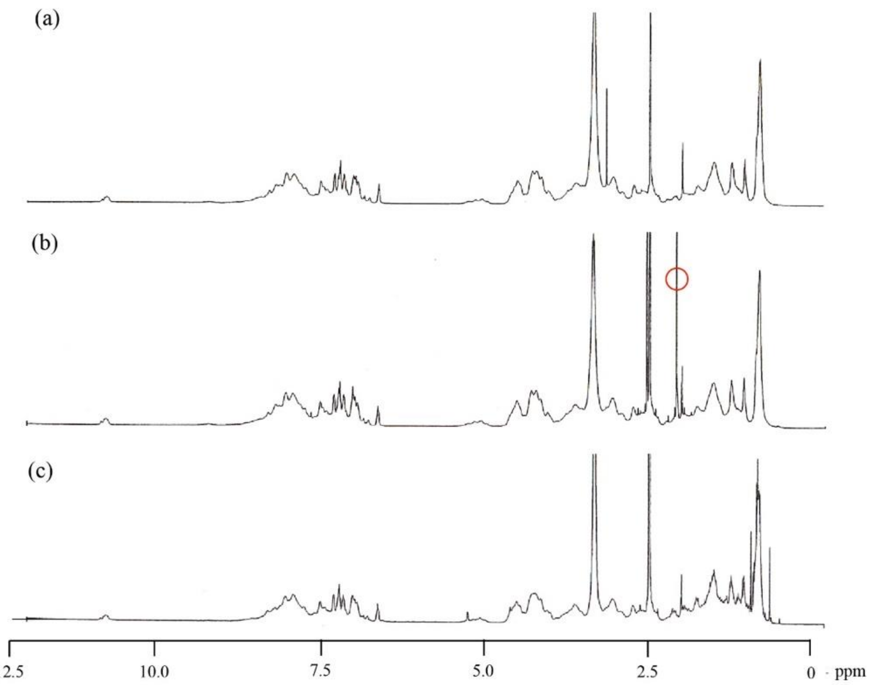

Figure S1. ^{1}H NMR spectra of each lysozyme in DMSO-d_6 at 25 °C. (**a**) Lysozyme, (**b**) CHLysozyme, and (**c**) physical mixture of lysozyme and cholesterol. Lysozyme and CHLysozyme concentration: 21.4 mg/mL; physical mixture of lysozyme and cholesterol (lysozyme [mol]/cholesterol [mol] = 1/12) concentration: 21.4 mg/mL.

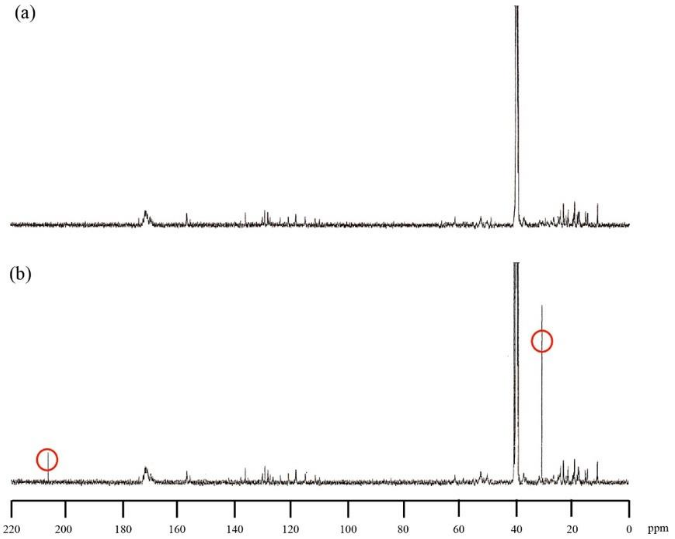

Figure S2. ^{13}C NMR spectra of lysozyme and CHLysozyme in DMSO-d6 at 25 °C. (**a**) Lysozyme, and (**b**) CHLysozyme. Lysozyme and CHLysozyme concentration: 21.4 mg/mL.

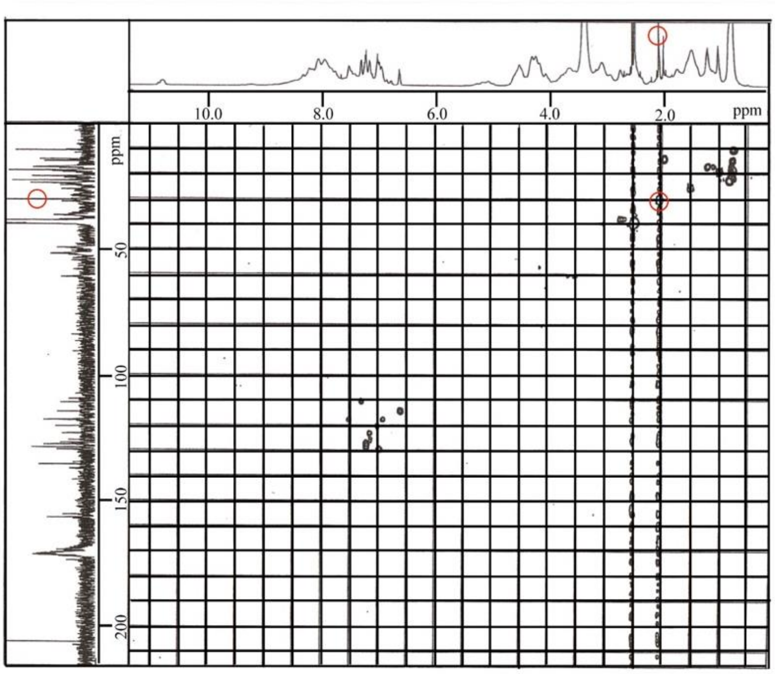

Figure S3. ^1H-^{13}C HMQC NMR spectrum of CHLysozyme in DMSO-d6 at 25 °C. CHLysozyme concentration: 21.4 mg/mL.

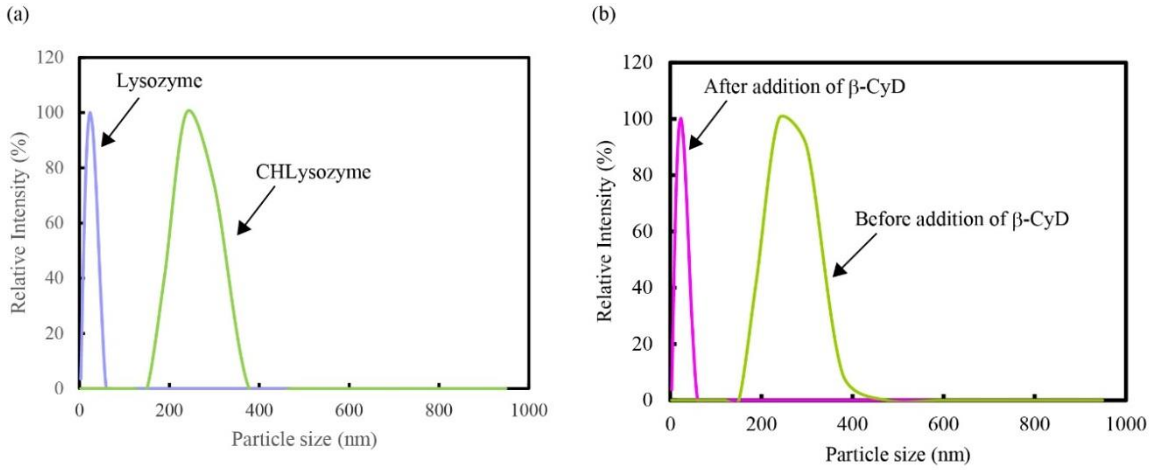

Figure S4. Particle size distribution of each lysozyme (**a**) and that of CHLysozyme before and after addition of β-cyclodextrin (β-CyD) (**b**). Lysozyme and CHLysozyme concentration: 1.0 mg/mL; β-CyD concentration: 0.2×10^{-3} mol/L. Preparation of the solution spiked with β-CyD: 0.1 mL of 0.2×10^{-3} mol/L β-CyD was added to 3.0 mL of CHLysozyme solution and the solution was analyzed 3 h post-preparation.

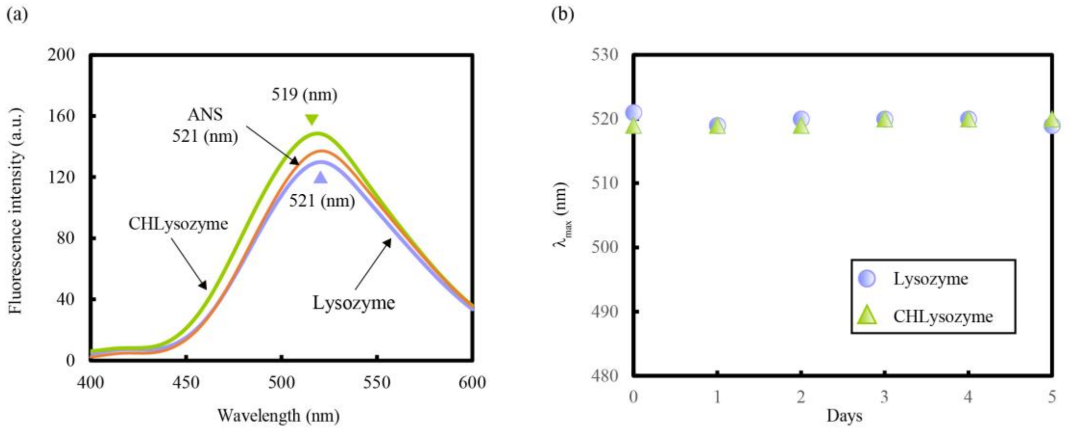

Figure S5. Fluorescence measurements of ANS alone and ANS with each lysozyme at 25 °C. (**a**) Fluorescence emission spectra of ANS alone and ANS with lysozyme and CHLysozyme at 25 °C. ANS concentration: 1.0 × 10^{-4} mol/L; lysozyme and CHLysozyme concentration: 0.02 mg/mL. Excitation wavelength: 365 nm. Gain value of fluorescence spectrometer: medium. (**b**) Change in λ_{max} of ANS with lysozyme and CHLysozyme against time at 25 °C. ANS concentration: 1.0 × 10^{-4} mol/L; lysozyme and CHLysozyme concentration: 0.02 mg/mL. Excitation wavelength: 365 nm. Gain value of fluorescence spectrometer: medium.

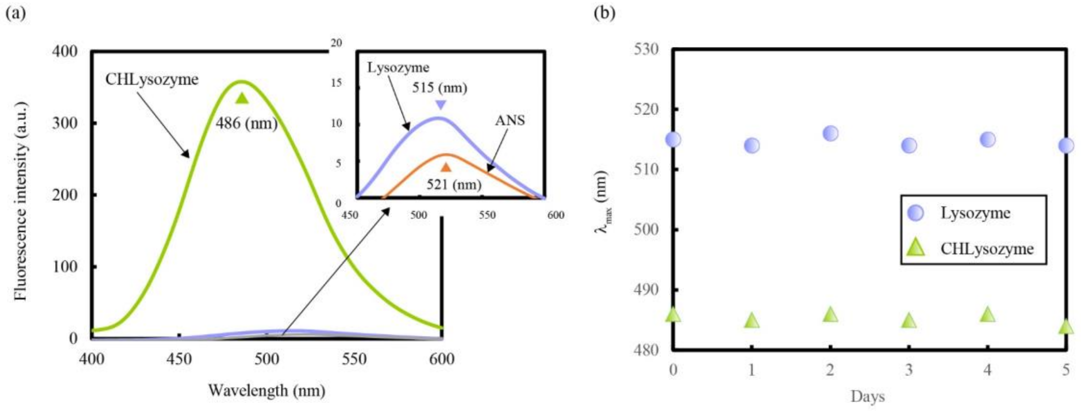

Figure S6. Fluorescence measurements of ANS alone and ANS with each lysozyme at 25 °C. (**a**) Fluorescence emission spectra of ANS alone and ANS with lysozyme and CHLysozyme at 25 °C. ANS concentration: 1.0 × 10^{-4} mol/L, lysozyme and CHLysozyme concentration: 2.0 mg/mL. Excitation wavelength: 365 nm. Gain value of fluorescence spectrometer: low. (**b**) Change in λ_{max} of ANS with lysozyme and CHLysozyme against time at 25 °C. ANS concentration: 1.0 × 10^{-4} mol/L; lysozyme and CHLysozyme concentration: 2.0 mg/mL. Excitation wavelength: 365 nm. Gain value of fluorescence spectrometer: low.

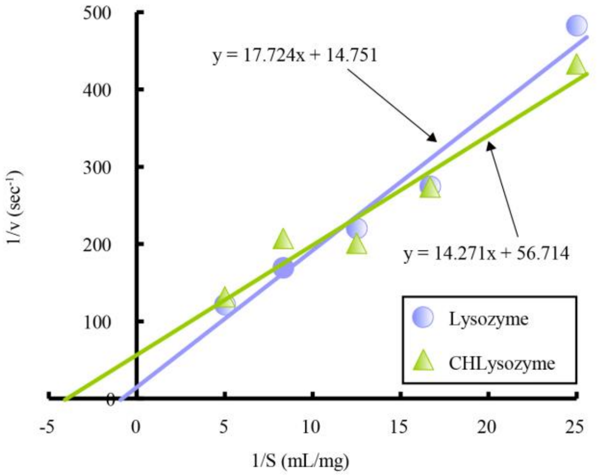

Figure S7. Lineweaver–Burk plots of lysozyme and CHLysozyme at 25 °C. Lysozyme and CHLysozyme concentration: 0.2 mg/mL; *Micrococcus lysodeikticus* concertation: 0.04–0.2 mg/mL.

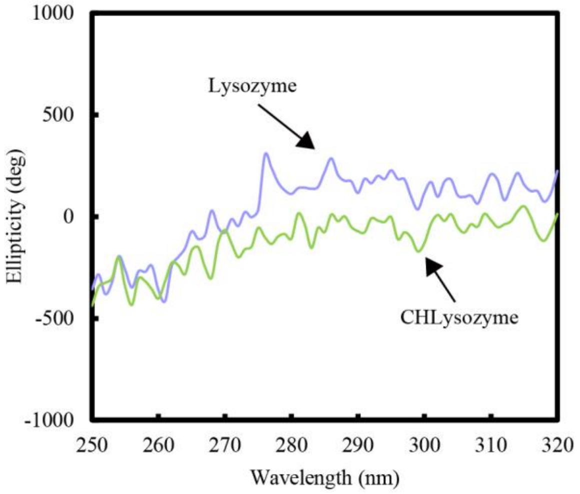

Figure S8. CD spectra of each lysozyme in near UV (250 to 320 nm) region at 25 °C after two days. Lysozyme and CHLysozyme concentration: 0.2 mg/mL.

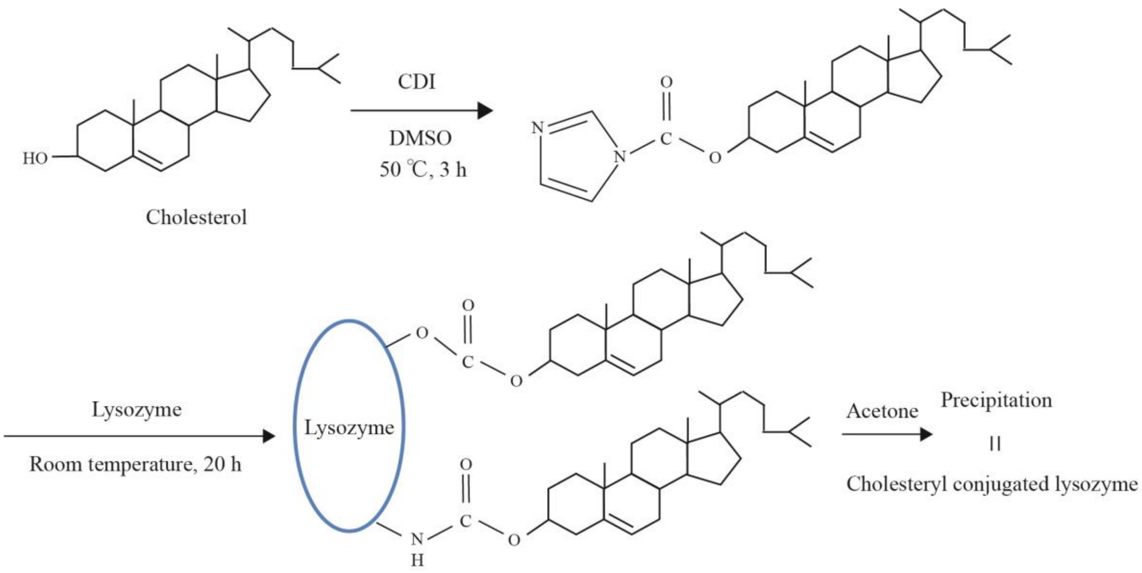

Figure S9. Synthetic procedure of CHLysozyme.
